# Genotypic characterisation of Avian paramyxovirus type-1 viruses isolated from aquatic birds in Uganda

**DOI:** 10.4102/ojvr.v85i1.1510

**Published:** 2018-06-25

**Authors:** Agnes Wanyana, Kizito K. Mugimba, Omony J. Bosco, Halid Kirunda, Jessica L. Nakavuma, Angélique Teillaud, Mariette F. Ducatez, Denis K. Byarugaba

**Affiliations:** 1 College of Veterinary Medicine, Makerere University, Uganda; 2 National Livestock Resources Research Institute, Tororo, Uganda; 3 Interactions Hôtes-Agents Pathogènes, Université de Toulouse, France; 4 École Nationale Vétérinaire de Toulouse, Toulouse, France

## Abstract

Avian paramyxovirus type-1 (APMV-1) viruses of the lentogenic pathotypes are often isolated from wild aquatic birds and may mutate to high pathogenicity when they cross into poultry and cause debilitating Newcastle disease. This study characterised AMPV-1 isolated from fresh faecal droppings from wild aquatic birds roosting sites in Uganda. Fresh faecal samples from wild aquatic birds at several waterbodies in Uganda were collected and inoculated into 9–10-day-old embryonated chicken eggs. After isolation, the viruses were confirmed as APMV-1 by APMV-1-specific polymerase chain reaction (PCR). The cleavage site of the fusion protein gene for 24 representative isolates was sequenced and phylogenetically analysed and compared with representative isolates of the different APMV-1 genotypes in the GenBank database. In total, 711 samples were collected from different regions in the country from which 72 isolates were recovered, giving a prevalence of 10.1%. Sequence analysis of 24 isolates revealed that the isolates were all lentogenic, with the typical ^111^GGRQGR’L^117^ avirulent motif. Twenty-two isolates had similar amino acid sequences at the cleavage site, which were different from the LaSota vaccine strain by a silent nucleotide substitution T357C. Two isolates, NDV/waterfowl/Uganda/MU150/2011 and NDV/waterfowl/Uganda/MU186/2011, were different from the rest of the isolates in a single amino acid, with aspartate and alanine at positions 124 and 129, respectively. The results of this study revealed that Ugandan aquatic birds indeed harbour APMV-1 that clustered with class II genotype II strains and had limited genetic diversity.

## Introduction

Avian paramyxovirus type 1 (APMV-1) belongs to the genus *Avulavirus*, in the family Paramyxoviridae, and is responsible for causing Newcastle disease (ND), a highly infectious disease for poultry (Alexander 2003). These viruses are usually grouped according to the pathotype based on the severity of the disease they may cause: velogenic (high virulence), mesogenic (mild virulence) and lentogenic (low virulence) viruses. The genetic basis of virulence is defined by the amino acid residues at the cleavage site of the fusion protein precursor (F_o_) (Aldous & Alexander 2001). The more virulent pathotypes that cause ND in poultry have multiple basic amino acids (at least three arginine or lysine residues between residues 113 and 116) with a phenylalanine at position 117 of the C-terminus of the F2 protein (also the N-terminus of the F1 protein) that make them cleavable by many proteases within the host tissues (Aldous & Alexander 2001; Aldous et al. 2003). The avirulent strains have fewer basic amino acid residues, with a leucine residue at position 117 of the F-protein. This portion of the cleavage site of the F-protein has been used not only for pathotyping APMV-1 viruses but also for genotyping the different strains (Aldous et al. 2003) among other typing methods that classify these strains into genotypes and lineages (Kim et al. 2007; Snoeck et al. 2009). Most of the genotyping methods classify these viruses into two classes, I and II, and further subdivide them into different genotypes and clades. The most recent classification has utilised the full F gene to classify these viruses further (Diel et al. 2012; Snoeck et al. 2013). The class II viruses are the most commonly reported and studied viruses and are associated with disease in poultry, pet and wild birds.

The role of APMV-1 recovered from wild birds has been alluded to in the epidemiology of ND in domestic poultry and aquatic birds in particular are thought to be the natural reservoirs (Jorgensen et al. 2004). Avirulent strains have particularly been consistently recovered from aquatic birds and their potential to mutate to virulent form upon passage in poultry has been confirmed (Shengqing et al. 1997; Takakuwa et al. 1998).

The migration of wild bird populations along various migration pathways across the world constitutes a serious threat to possible spread of these viruses and pause a risk of transmission to domestic poultry (Zarkov et al. 2005). Studies have already shown similarities between strains recovered from aquatic birds and shorebirds with those isolated from live-bird markets in some parts of the world (Kim et al. 2007), further confirming this threat. We have previously demonstrated that virulent APMV-1 strains circulate in live-bird markets in Uganda in apparently healthy birds (Byarugaba et al. 2014). However, despite the massive number of migratory and resident birds that rest along several waterbodies in Uganda, no studies have been undertaken to understand whether these birds harbour APMV-1. With the biggest proportion of the poultry production sector in Uganda being backyard, there is a high risk of transmission of the APMV-1 from aquatic birds into the poultry population that may result in economic losses to both the small-scale poultry farmers and the country. This study sought to establish if APMV-1 strains circulate in aquatic birds in Uganda and how they compare genetically to others elsewhere.

## Materials and methods

### Sample collection

Fresh faecal samples were collected from aquatic bird (which includes all aquatic birds and waterfowl) roosting sites along various waterbodies across the country using sterile dacron swabs into cryovials containing virus transport medium supplemented with antibiotics (isotonic phosphate buffered saline, 2000 U/mL penicillin, 2 mg/mL streptomycin, 50 *μ*g/mL gentamycin, 50 U/mL nystatin and 0.5% bovine serum albumin). The samples were stored and transported in dry shippers until delivered to the laboratory where they were stored at -80 ^o^C until further use. A total of 711 samples were collected from various sites including Musambwa Island, Makanaga Bay, Lutembe Bay, Mabamba Bay, Nakiwogo landing site, Samuka Island, Macdonald Bay, Doho Rice Scheme, Lake Bisina Island, Murchison Falls National Park, Queen Elizabeth National Park, and Kibimba Dam Rice Scheme.

### Virus isolation

The samples were inoculated (in triplicate) by the allantoic route into 9–10-day embryonated chicken eggs for virus isolation according to the World Organisation for Animal Health (OIE) Manual of Standards for Diagnostic Tests and Vaccines (OIE 2008). Allantoic fluid was harvested 3 days post-inoculation and subsequently tested for haemagglutination (HA) using 1% chicken erythrocytes and haemagglutination inhibition (HI) with in-house–generated polyclonal anti-APMV-1 sera as described (OIE 2008). The HI-positive samples were subsequently confirmed by polymerase chain reaction (PCR).

### Confirmation by reverse transcription-polymerase chain reaction

Viral ribonucleic acid (RNA) was extracted from all the HI-positive samples using the QIAamp Viral RNA mini kit (Qiagen, Germantown, MD, USA) according to the manufacturer’s instructions. Polymerase chain reaction was performed with a Qiagen one-step reverse transcription-polymerase chain reaction (RT-PCR) kit (Qiagen, USA) according to the manufacturer’s instructions, with the following APMV-1 primers FOP1: 5’ TACACCTCATCCCAGACAGGGTC 3’ (nucleotide position, 158–177) and FOP2: 5’ AGGCAGGGGAAGTGATTTGTGGC 3’ (nucleotide position, 493–513) according to Kho et al. (2000). The primers were used for the amplification of a 356 bp region corresponding to the cleavage activation site of F gene of APMV-1. The RT-PCR was performed in a 25 *µ*L reaction volume containing 5 *µ*L of 5X RT-PCR buffer, 11 *µ*L of RNAse-free H_2_O, 1 *µ*L of 10 mmol/L dNTPs, 1.5 *µ*L of 10 nmol/L of each primer, 2 *µ*L of 50 mM MgCl_2_, 1 *µ*L of enzyme mix (Taq DNA polymerase and reverse transcriptase) and 2 *µ*L of viral RNA extract. Amplification was carried out in an Applied Biosystems Veriti 96-well thermocycler with a single reverse transcription (RT) step of 50 ºC for 30 min, a denaturation step of the RT (95 ºC) for 15 min, followed by 40 cycles with 30 s denaturation at 95 ºC, 30 s of primer annealing at 58 ºC, 1 min of extension at 72 ºC and a final extension for 10 min at 72 ºC. The samples (including a known positive control) were then separated on a 1% agarose gel with a 100-bp marker.

### Sequencing

A total of 24 representative isolates were selected for sequencing of the partial cleavage site of the fusion gene. Out of the 72 isolates, we selected 24 isolates chosen proportionally from each site including 7/23 from Musambwa, 8/22 from Lutembe, 5/8 from Makanaga, 1/6 from Samuka, 1/7 from Nakiwogo and 1/6 from Queen Elizabeth National Park. The fragments were run on a 1% agarose gel, excised from the gel and purified with QIAquick PCR Purification Kits (Qiagen, USA) according to the manufacturer’s recommendations. Sanger sequencing was carried out on the purified PCR products using the same primers that were used for the PCR. Sequencing was performed on a 3130XL Applied Biosystems capillary sequencer at the Plateau de Génomique GeT-Purpan, UDEAR UMR 5165 CNRS/UPS, CHU PURPAN, Toulouse, France.

### Phylogenetic analysis

The Basic Local Alignment Search Tool (BLAST) was used to find similar F gene sequences for APMV-1 in the Genbank. Sequences covering the cleavage site of the fusion gene representing all 18 genotypes of APMV-1 recently described by Diel et al. (2012), including all the Ugandan and East African sequences, were retrieved. The sequences were aligned together with the Ugandan sequences generated in this study using Clustal W and edited using Bioedit Software version 5.0.9 (Hall 1999). Phylogenetic analysis was performed using the MEGA version 5.05 program (Tamura et al. 2011) with the neighbour-joining (NJ) Kimura 2-parameter method and 1000 bootstrap replicates. The amino acids around the fusion protein cleavage site were compared to representative sequences from each of the genotypes. Vaccine strains – LaSota, accession number: JF950510; Hitchner BI, accession number: JN872151 and I-2, accession number AY935499 – were also included in the analysis.

### Availability of data and materials

The sequences of the cleavage site of the isolates analysed in this study were deposited in GenBank with accession numbers LT549451, LT549452 and LT549453. These include NDV138/aquatic birds/Uganda/2011 representing the 22 isolates with identical sequences and NDV150/aquatic birds/Uganda/2011 and NDV186/aquatic birds/Uganda/2011 as indicated in the phylogenetic tree legend in Figure 2.

### Ethics

This study was approved by the College of Veterinary Medicine Animal Resources and Biosecurity Higher Degrees Research Committee and Uganda National Council of Science and Technology (Approval # HS 776).

## Results

### Occurrence of Avian paramyxovirus type-1

From the 711 samples collected, 72 isolates were recovered. The prevalence at each site ranged from 0% to 36% and the average was estimated as 10.1% by HI (Table 1). No isolates were recovered from Doho Rice Scheme, Lake Bisina, Mabamba, Murchision Falls and Kibimba Dam Rice Scheme. Six isolates were obtained from Queen Elizabeth National Park, 22 from Lutembe, 8 from Makanaga, 23 from Musambwa, 7 from Nakiwogo and 6 from Samuka (Table 1). Musambwa Island and Lutembe Bay provided the highest number of isolates, with Queen Elizabeth and Nakiwogo providing the lowest number of isolates. Prevalence was highest in Musambwa, Lutembe and Makanaga. At the sites, the most common bird species were the grey-headed gull, white-winged tern, gull-billed tern, long-tailed cormorants, great cormorant, Egyptian geese and others as indicated in Table 1.

**FIGURE 1:**
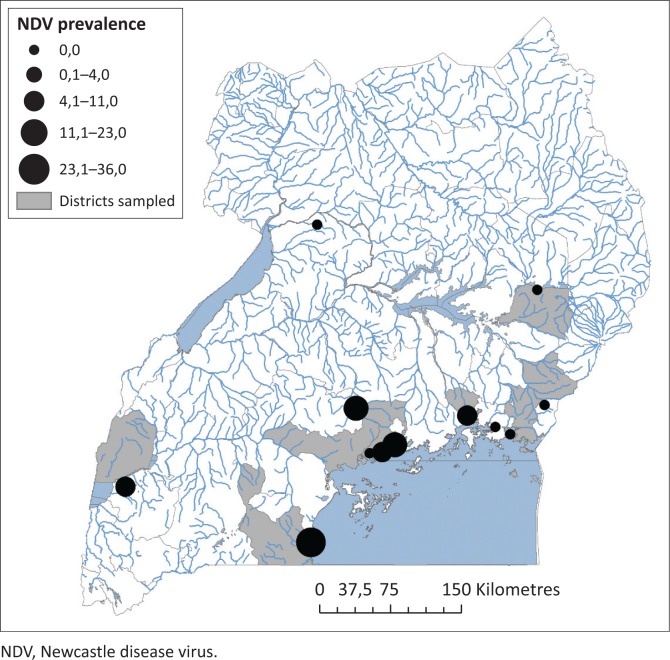
Prevalence distribution of Avian paramyxovirus-1 in aquatic birds roosting sites in Uganda.

**TABLE 1:** Occurrence of Avian paramyxovirus type-1 by site among migratory aquatic birds.

Sampling site	GPS coordinates (longitude, latitude)	Estimates of total bird		Most predominant bird species at site	Occurrence	
		Count	Species		%	N†
Samuka Island	33.275185, 0.399174	420	12	Long-tailed Cormorant, Little Egret, African Open-billed Stork, Grey-headed Gull, Black-headed Heron,	9.2	6/65
Makanaga wetland	32.655487, 0.091581	2500	26	Slender-billed Gull, Gull-billed Tern, African Jacana, Long-tailed Cormorant, Yellow-billed Duck, Grey-headed Gull	17.4	8/46
Macdonald Bay	33.762360, 0.143766	760	20	Long-tailed Cormorant, Grey-headed Gull, White-winged Tern Little Egret, Greater Cormorant, Egyptian Goose, Slender-billed Gull,	0.0	0/44
Doho Rice Scheme	33.704681, 0.135527	560	19	Little Egret, African Open-billed Stork, Black Crake, Black-headed Heron, Long-tailed Cormorant, Yellow-billed Stork	0.0	0/29
Musambwa Island	32.439880, -1.750159	650	32	Grey-headed Gull, Long-tailed Cormorant, Greater Cormorant, Sacred Ibis, Pink-backed Pelican, Black Crake, Egyptian Goose	35.9	23/64
Lutembe wetland	32.581716, 0.155726	1670	30	White-winged Tern, Grey-headed Gull, Yellow-billed Duck, Long-toed Plover, Gull-billed Tern, Long-tailed Cormorant	22.0	22/100
Murchison Falls NP	31.482525, 2.201648	1560	33	Pied Kingfisher, African Jacana, African Darter, Hadada Ibis, Egyptian Goose, Cattle Egret, African Fish Eagle	0.0	0/79
Nakiwogo –L. Victoria	32.365894, -0.047604	8900	42	White-winged Tern, Long-tailed Cormorant, Grey-headed Gull, Slender-billed Gull, Little Egret, Greater Cormorant, Egyptian Goose	10.7	7/65
Queen Elizabeth NP	29.911222, -0.222585	4200	35	Pied Kingfisher, Greater Cormorant, Egyptian Goose, Hamerkop, Yellow-billed Stork, Black Crake	4.4	6/137
L. Bisina	34.050064, 1.342055	50	12	Long-tailed Cormorant, African Open-billed Stork, White-winged Tern, Green-backed, Heron, Long-toed Plover, Black Crake	0.0	0/19
Mabamba wetland	32.430611, -0.027916	220	20	Long-toed Plover, African Jacana, Pied Kingfisher,White-faced Whistling Duck, Malachite Kingfisher, Common Squacco, Heron, Long-tailed Cormorant	0.0	0/40
Kibimba Dam Rice Scheme	33.776093, -0.012789	1800	28	Egrets, African Open-billed Stork, Black-headed Heron, Terek Sandpiper, Wood Sandpiper, Long-tailed Cormorant	0.0	0/23

GPS, global positioning system.

† , number of positive over total samples collected.

### Pathotypes of Avian paramyxovirus type-1 in Ugandan aquatic birds

The deduced amino acid sequences of the F gene cleavage site were used to determine the pathotypes and are shown in Table 2. All 24 isolates sequenced had a lentogenic motif of ^111^GGRQGR’L^117^ characteristic of the avirulent strains. However, isolates NDV150/waterfowl/Uganda/2011 and NDV186/waterfowl/Uganda/2011 were different from the rest of the 22 isolates in a single amino acid; aspartate and alanine at positions 124 and 129, respectively. The rest of the 22 isolates were identical at all positions.

**TABLE 2:** F-Protein cleavage site motif of the Newcastle disease viruses sequenced in this study.

No.	Isolate name	Date of isolation	Site/location	F gene cleavage site (111-117) sequence	Pathogenicity
1	APMV-1/waterfowl/Uganda/MU122/2011	17/07/2011	Queen Elizabeth NP/Kasese	GGRQGR’**L**	LENTOGENIC
2	APMV-1/waterfowl/Uganda/MU125/2011	11/07/2011	Lutembe Bay/Entebbe	GGRQGR’**L**	LENTOGENIC
3	APMV-1/waterfowl/Uganda/MU126/2011	11/07/2011	Lutembe Bay/Entebbe	GGRQGR’**L**	LENTOGENIC
4	APMV-1/waterfowl/Uganda/MU129/2011	11/07/2011	Lutembe Bay/Entebbe	GGRQGR’**L**	LENTOGENIC
5	APMV-1/waterfowl/Uganda/MU130/2011	11/07/2011	Lutembe Bay/Entebbe	GGRQGR’**L**	LENTOGENIC
6	APMV-1/waterfowl/Uganda/MU131/2011	11/07/2011	Lutembe Bay/Entebbe	GGRQGR’**L**	LENTOGENIC
7	APMV-1/waterfowl/Uganda/MU132/011	11/07/2011	Lutembe Bay/Entebbe	GGRQGR’**L**	LENTOGENIC
8	APMV-1/waterfowl/Uganda/MU137/2011	11/07/2011	Lutembe Bay/Entebbe	GGRQGR’**L**	LENTOGENIC
9	APMV-1/waterfowl/Uganda/MU138/2011	11/07/2011	Lutembe Bay/Entebbe	GGRQGR’**L**	LENTOGENIC
10	APMV-1/waterfowl/Uganda/MU149/2011	25/09/2011	Makanaga Bay/Mpigi	GGRQGR’**L**	LENTOGENIC
11	APMV-1/waterfowl/Uganda/MU150/2011	25/09/2011	Makanaga Bay/Mpigi	GGRQGR’**L**	LENTOGENIC
12	APMV-1/waterfowl/Uganda/MU151/2011	25/09/2011	Makanaga Bay/Mpigi	GGRQGR’**L**	LENTOGENIC
13	APMV-1/waterfowl/Uganda/MU152/2011	25/09/2011	Makanaga Bay/Mpigi	GGRQGR’**L**	LENTOGENIC
14	APMV-1/waterfowl/Uganda/MU154/2011	25/09/2011	Makanaga Bay/Mpigi	GGRQGR’**L**	LENTOGENIC
15	APMV-1/waterfowl/Uganda/MU159/2011	12/09/2011	Musambwa Island/Rakai	GGRQGR’**L**	LENTOGENIC
16	APMV-1/waterfowl/Uganda/MU162/2011	12/09/2011	Musambwa Island/Rakai	GGRQGR’**L**	LENTOGENIC
17	APMV-1/waterfowl/Uganda/MU165/2011	12/09/2011	Musambwa Island/Rakai	GGRQGR’**L**	LENTOGENIC
18	APMV-1/waterfowl/Uganda/MU167/2011	12/09/2011	Musambwa Island/Rakai	GGRQGR’**L**	LENTOGENIC
19	APMV-1/waterfowl/Uganda/MU170/2011	12/09/2011	Musambwa Island/Rakai	GGRQGR’**L**	LENTOGENIC
20	APMV-1/waterfowl/Uganda/MU171/2011	12/09/2011	Musambwa Island/Rakai	GGRQGR’**L**	LENTOGENIC
21	APMV-1/waterfowl/Uganda/MU172/2011	12/09/2011	Musambwa Island/Rakai	GGRQGR’**L**	LENTOGENIC
22	APMV-1/waterfowl/Uganda/MU173/2011	12/09/2011	Musambwa Island/Rakai	GGRQGR’**L**	LENTOGENIC
23	APMV-1/waterfowl/Uganda/MU181/2011	12/03/2012	Nakiwogo/Entebbe	GGRQGR’**L**	LENTOGENIC
24	APMV-1/waterfowl/Uganda/MU186/2011	23/09/2011	Samuka Island/ Jinja	GGRQGR’**L**	LENTOGENIC

Note: Twenty-four representative APMV-1 isolates were sequenced. All the isolates showed the lentogenic amino acid motif ^111^GGRQGR’**L**^117^.

APMV-1, Avian paramyxovirus type-1.

**FIGURE 2:**
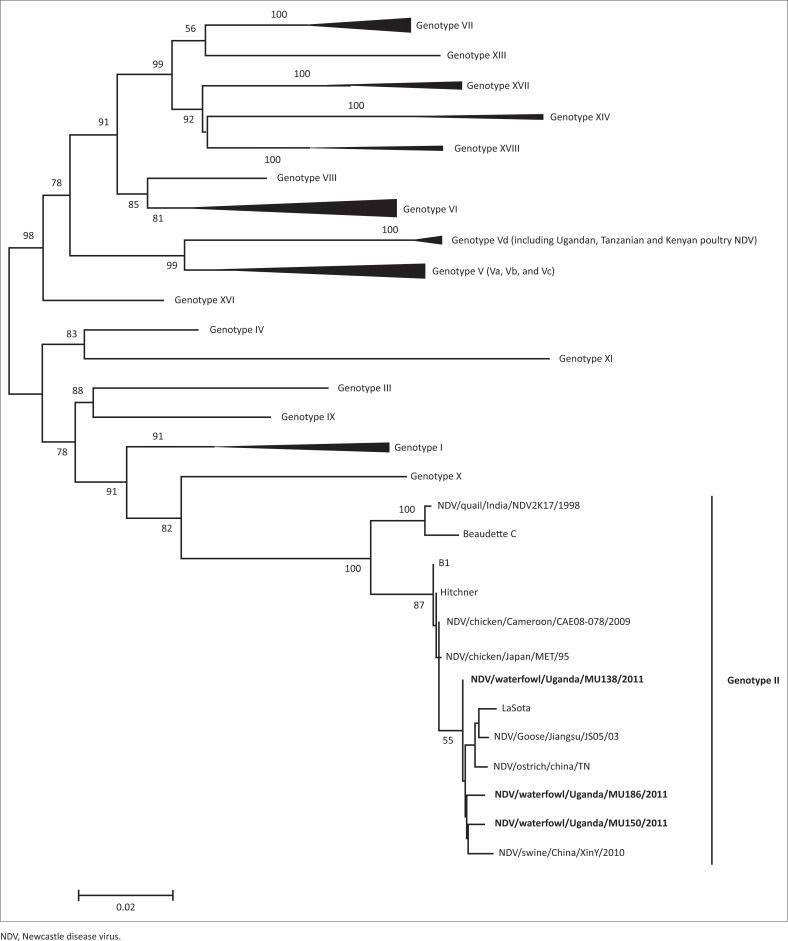
Phylogenetic analysis of partial F nucleic acid sequences of Avian paramyxovirus type-1. The tree was generated by a neighbour-joining algorithm, and alignments were bootstrapped 1000 times (only bootstraps > 50 are shown). The strain names were edited to include origin of isolates where needed. Genotypes are marked on the right. Representative isolates from the 18 recently described genotypes (according to the new proposed classification by Diel et al. (2012) are included and collapsed together, except for the genotype II where the aquatic birds isolates from this study belong.

**Phylogenetic analysis:** To determine the phylogenetic relationships between Ugandan aquatic birds’ isolates, other Ugandan strains from poultry and the rest of the world, the sequences of the 201-bp hypervariable region of the F gene were compared to the corresponding region of viruses available in GenBank. Results from the phylogenetic analysis clustered our isolates with genotype II strains which had also been historically described as genotype II or lineage 2. They were different from the recently isolated strains from poultry in Uganda which belonged to genotype V as shown in Figure 2.

## Discussion

This study is the first to isolate and characterise APMV-1 from aquatic birds in Uganda. Most work on APMV-1 in many parts of the world has focused on poultry, where occurrence of both virulent strains and lentogenic strains of class II has been reported with limited studies in wild birds (de Almeida et al. 2013; Miller, Decanini & Afonso 2010). Virulent APMV-1 is a common cause of infections in birds and more than 230 bird species have been reported to be susceptible in experimental infections (USDA/APHIS/WS 2016) including those we found at the sites where we collected our samples, such as the cormorants. The economic impact of ND on the poultry industry in Uganda and elsewhere is significant both in the backyard and commercial flocks. Little is known about the APMV-1 strains circulating in wild birds, their evolution and their role in the epidemiology of the disease. Czeglédi et al. (2006) speculated that class I and class II genotype I are ancestral representatives of APMV-1 maintained by their natural hosts, the wild waterfowl.

In the present study, we demonstrated the occurrence of APMV-1 among aquatic birds in Uganda with a 10.1% prevalence, which was higher than that reported (2.1%) in other studies in Africa (de Almeida et al. 2013). A few studies in Uganda have shown the occurrence of virulent APMV-1 strains (genotype V) that circulate among Ugandan poultry and live-bird markets (Byarugaba et al. 2014; Otim et al. 2004). Unlike in our study where the 24 sequenced isolates were clustered with genotype II, other studies of APMV-1 in wild birds in Africa and elsewhere have demonstrated the presence of both genotype I and II strains in wild birds (Miguel et al. 2013; Snoeck et al. 2009), with a possible involvement of inter- or intracontinental bird migration. Others have reported lentogenic APMV-1 in wild birds outside Africa (Banura et al. 2013; Krapez et al. 2010; Lindh et al. 2012; Stanislawek et al. 2000; Takakuwa et al. 1998). Some of these studies indicate that wild birds may play a role as a potential source of virulent APMV-1 for poultry. Moreover, it is suggested that velogenic APMV-1 might arise from lentogenic APMV-1 in nature through point mutations in the F-protein cleavage site, making them virulent for poultry (de Leeuw et al. 2003; Takakuwa et al. 1998). Evolution of APMV-1 has continuously posed threats for emergence of new virulent strains and challenges for diagnosis of ND (Cattoli et al. 2010; Miller et al. 2010). Toyoda et al. (1989) suggested that different strains of APMV-1 evolve through various degrees of accumulation of point mutations rather than gene exchange by recombination. Such emerging virulent strains related to lentogenic strains (antigenically and genetically) have been suspected to have caused outbreaks in Ireland in 1990 (Toyoda et al. 1989; Alexander et al. 1992) and in Australia in 1998–2000 (Gould et al. 2001; Westbury 2001).

Phylogenetic analysis of APMV-1 isolates isolated in the current study showed that the partial F gene sequences clustered with those of genotype II viruses. This is consistent with previous reports of a predominance of genotype II viruses in wild birds (Hoque et al. 2012). The earlier revelation that the highly virulent strains could evolve from viruses of low virulence by mutation (Gould et al. 2001; Westbury 2001) underscores the significance of more detailed genomic studies to ascertain possible epidemiological linkages of strains circulating in wild birds and poultry. Although velogenic strains of APMV-1 have been isolated from wild birds suggesting an epidemiological link with strains in poultry (Huovilainen et al. 2001; Jorgensen et al. 2004; Liu et al. 2008; Snoeck et al. 2013; Zarkov et al. 2005; Zhu et al. 2010), the current study did not recover any virulent pathotype. Our recent studies on APMV-1 in domestic poultry revealed a separate genotype (V) (Byarugaba et al. 2014). This does not mean virulent strains may not occur in aquatic birds in the country and therefore more extensive molecular epidemiological and routine monitoring for APMV-1 in aquatic birds is important for early detection to prevent any possible spillover into domestic poultry. Such detailed genomic studies will elucidate the exact role of wild birds in the ecology and epidemiology of APMV-1 in poultry and inform control strategies.
